# Somatic Mosaicism in Cases with Small Supernumerary Marker Chromosomes

**DOI:** 10.2174/138920210793176029

**Published:** 2010-09

**Authors:** Thomas Liehr, Tatyana Karamysheva, Martina Merkas, Lukrecija Brecevic, Ahmed B. Hamid, Elisabeth Ewers, Kristin Mrasek, Nadezda Kosyakova, Anja Weise

**Affiliations:** 1 Jena University Hospital, Institute of Human Genetics and Anthropology, Jena, Germany; 2 Institute for Cytology and Genetics, Nowosibirsk, Russian Federation; 3 School of Medicine Zagreb University, Croatian Institute for Brain Research, Zagreb, Croatia

**Keywords:** Mosaic, small supernumerary marker chromosomes (sSMC), genotype-phenotype correlation.

## Abstract

Somatic mosaicism is something that is observed in everyday lives of cytogeneticists. Chromosome instability is one of the leading causes of large-scale genome variation analyzable since the correct human chromosome number was established in 1956. Somatic mosaicism is also a well-known fact to be present in cases with small supernumerary marker chromosomes (sSMC), i.e. karyotypes of 47,+mar/46. In this study, the data available in the literature were collected concerning the frequency mosaicism in different subgroups of patients with sSMC. Of 3124 cases with sSMC 1626 (52%) present with somatic mosaicism. Some groups like patients with Emanuel-, cat-eye- or i(18p)- syndrome only tend rarely to develop mosaicism, while in Pallister-Killian syndrome every patient is mosaic. In general, acrocentric and non-acrocentric derived sSMCs are differently susceptible to mosaicism; non-acrocentric derived ones are hereby the less stable ones. Even though, in the overwhelming majority of the cases, somatic mosaicism does not have any detectable clinical effects, there are rare cases with altered clinical outcomes due to mosaicism. This is extremely important for prenatal genetic counseling. Overall, as mosaicism is something to be considered in at least every second sSMC case, array-CGH studies cannot be offered as a screening test to reliably detect this kind of chromosomal aberration, as low level mosaic cases and cryptic mosaics are missed by that.

## SMALL SUPERNUMERARY MARKER CHROMOSOMES (sSMC)

In 1956, the exact chromosomal number in humans was established [1]. Since then it was possible to delineate numerical chromosomal aberrations in any body tissue where chromosomes could be prepared from, including clinical [2] and tumor cases [3]. After the introduction of molecular cytogenetics [4-7], it became even possible to analyze numerical chromosomal aberrations in non-dividing cells [8]. By that also low-level chromosomal aberrations could be detected in tumor [9-13], various clinical [14-18] and neuronal diseases [19-27], embryonic tissues [28-32] and different tissue types [9, 13, 15, 33-35]. Overall it can be stated that chromosome instability is one of the main causes of large-scale genome variation [36-39]. For review of cytogenetic and molecular cytogenetics see Refs. [4-5, 40]. 

Small supernumerary marker chromosomes (sSMC) are reported in 0.043% of newborn infants, 0.077% of prenatal cases, 0.433% of mentally retarded patients and 0.171% of subfertile people [41]. They are defined as structurally abnormal chromosomes that cannot be identified or characterized unambiguously by conventional banding cytogenetics alone, and are generally equal in size or smaller than a chromosome 20 of the same metaphase spread; sSMC can either be present additionally in (1) an otherwise normal karyotype, (2) a numerically abnormal karyotype (like Turner- or Down-syndrome) or (3) a structurally abnormal but balanced karyotype with or without ring chromosome formation [42]. sSMCs are normally detected by banding cytogenetics in mentally retarded patients, in subfertile persons or during prenatal diagnosis and particularly prenatally ascertained ones, are not easy to correlate with a clinical outcome. It is known that ~30% of sSMCs are derived from chromosome 15; ~11% are i(12p) = Pallister-Killian, ~10% are der(22)-Emanuel-,~7% are inv dup (22)-cat-eye- and ~6% are i(18p)-syndrome associated sSMC [42].

sSMC are for several reasons still a problem in clinical cytogenetics: (i) they are too small to be characterized for their chromosomal origin by traditional banding techniques and require molecular (cytogenetic) techniques for their identification [41]; (ii) apart from the correlation of about one-third of the sSMC cases with a specific clinical picture, as mentioned above, most of the sSMCs have not been correlated with clinical syndromes, even though progress was achieved, recently [43, 44]; (iii) sSMC can be harmful due to different mechanisms like induction of genomic imbalance and/or uniparental disomy [42]; (iv) also sSMC can be found just by chance and cannot be correlated with the clinical problems of a patient [44]; finally (v) the percentage in which an sSMC is present can, but must not have an influence on the clinical outcome [42-44]. 

Here we focus on the latter mentioned problem – the regularly appearing somatic mosaicism in cases with an sSMC. 

Mosaicism in association with sSMC is a well-known fact. Crolla (1998) [45] summarized 144 sSMC cases excluding those derived from chromosomes 15 and 22, 78 of which (54%) showed mosaic karyotypes. To get a more detailed view on mosaicism in sSMC the following subgroups are focused separately below: cases with sSMC duplication and multiple sSMC, cases with four known ‘sSMC-syndromes’ Pallister-Killian-, i(18p)-, Emanuel-, and cat-eye-syndrome, cases with sSMC and Prader-Willi- and Angelman-syndrome, cases with an sSMC present in a structurally abnormal but balanced karyotype, neocentric sSMC cases and patients with numerically abnormal basic karyotypes. The remaining sSMC with a normal basic karyotype of 46 chromosomes plus an sSMC are the group of patients this review starts with.

## SOMATIC MOSAICISM IN sSMC PRESENT IN A NORMAL BASIC KARYOTYPE

According to Liehr (2010) [44] 731/1512 sSMC cases (52%) studied by cytogenetics are mosaic (see Table **1**). However, there is a strong difference between acrocentric and non-acrocentric derived sSMC: while 72% of acrocentric derived sSMC present no mosaic, 82% of non-acrocentric derived sSMCs are mosaic. 

The real grade and complexity of mosaicism seems to be even slightly higher as recently repeatedly cryptic mosaicism was detected in sSMC cases by molecular cytogenetics. There were either cases showing an sSMC in all studied metaphase spreads, however, interphase-FISH in uncultured cells showed a mosaic situation like in case 16-CW-2 [44]. More often it is found that more than one variant of an sSMC is present in different studied cells of a patient. As summarized in Table **2**, at least 5% of sSMC cases have, after a detailed molecular cytogenetic analysis, a more complex mosaicism than suggested after simple cytogenetic diagnostics. In 20% of these cases, unexpected complex somatic mosaicism was detectable where cytogenetics did not suggest any mosaicism, i.e. in cases 04-U-7, 08-W-p11.2/1-2, 11-O-p11.1/2-1, 11-U-9, 13-U-13, 15-W-q11.1+q11.2/1-1, 21-O-q11.21/1-1, 21-U-5, 22-O-q11.1/5-1, 22-O-q11.1/5-2, 0X-W-p11.?3/1-1, 0X-W-p11.21/1-1 [44]. Interestingly, acrocentric derived sSMC are by far more stable than non-acrocentric derived ones (2% versus 9%, Table **2**). 

Cryptic mosaicism appears as some sSMC tend to rearrange and/or be reduced in size during karyotypic evolution. This can lead to double ring formation or inverted duplication starting from a centric minute-shaped chromosome and in the end to the formation of different variants and a highly complex mosaic as some of the new variants can also be degraded in a subset of the studied cells [46].

In summary, somatic mosaics are to be expected in at least 50% of sSMC cases with normal basic karyotype. More complex mosaics can be met in up to 10% of the cases; however, the overall rate of mosaic cases is not significantly altered by cryptic mosaicism, while the genetic complexity of individual cases may be severely influenced.

## SOMATIC MOSAICISM IN CASES WITH sSMC DUPLICATION AND MULTIPLE sSMC

sSMC in a small subset of cases tend to duplicate, leading to a karyotype 48,+marx2 [42]. Up to now 64 such cases are reported [44] and 45% of those are derived from non-acrocentric chromosomes (Table **3**). While, cases with acrocentric derived sSMC tend to be by mosaic only in 54% of the cases, non-acrocentric derived ones are always mosaic with an exception of 1/29 reported patients (Table **3**). Thus, in sSMC duplication cases we find a similar situation as in cases with one single sSMC and a karyotype 47, +mar concerning mosaicism. 

Multiple sSMC cases [42] differ from sSMC duplication ones by the fact that the observed sSMC are not derived from the identical chromosome. Only 48 such cases are known by now [44], having between 2 and 7 sSMC of different origin, each; and all reported cases with multiple sSMC are mosaic. Formation of this rare cytogenetic condition is unclear, even though polysomic rescue or triploid rescue maybe suggested. As in most cases markedly chromosomal imbalances are induced by multiple sSMC presence, ~90-95% of them are correlated with clinical symptoms, irrespective of mosaicism status detectable in peripheral blood.

## SOMATIC MOSAICISM PRESENT IN THE FOUR KNOWN ‘sSMC-SYNDROMES’: PALLISTER-KILLIAN-, I(18P)-, EMANUEL-, AND CAT-EYE-SYNDROME

Somatic mosaicism is reported to different extents in four sSMC-related syndromes.

Patients suffering from Pallister-Killian-syndrome (PKS) due to the presence of an additional isochromosome 12p are known to have somatic mosaicism in practically every case. In peripheral blood the +(12p) tends to be lost either already during pregnancy or shortly after birth in practically all cells. In the alternatively studied skin fibroblasts, the sSMC derived from chromosome 12 is normally easily to detect in >70% to 100% of the cells [47]. However, besides a mosaic of cells with 46 and 47 chromosomes exceptional cases also with two different shapes of sSMC (12-Wpks-4, 12-Wpks-159, [44]) or two isochromosomes 12p (12-Wpks-174 [44]) are also reported. 

In isochromosome 18p syndrome mosaicism is rather rare. But also here exceptional cases are known having the full clinical phenotype but normal karyotype in some of the body cells (18-Wi-42, 18-Wi-153, 18-Wi-154, 18-Wi-157 [44]). In case 18-Wi-41 [44] the i(18p) was derived from the clinically normal mother; the latter had the i(18p) in only 4% of her peripheral blood cells. Also, an interesting case of somatic mosaicism is 18-Wi-158 [44] showing prenatally an i(18p) in 35% of the amnion cells but postnatal only normal cells in peripheral blood, being a normal child.

To the best of our knowledge no mosaic cases are known by now for Emanuel-syndrome (ES) [44]. Also in cat-eye-syndrome (CES) mosaicism is rather rare. sSMC derived from chromosome 22 having two different shapes were seen in CES (22-Wces-2 [44]) or minimal mosaicism with a normal cell line (22-Wces-3-03, 22-Wces-5, 22-Wces-5-119 [44]). 

Overall, somatic mosaicism is, compared to other sSMC derived from the corresponding chromosomes, over-represented in PKS (100% vs. 79%) and under-represented in i(18p) syndrome (4% vs. 67%), ES (0% vs. 32%) and CES (3% vs. 32%).

## SOMATIC MOSAICISM IN PRADER-WILLI- AND ANGELMAN-SYNDROME WITH sSMC

26 sSMC cases with Prader-Willi-syndrome (PWS) and 7 with Angelman-syndrome (AS) can be found in the literature [44]. 15 of these are PWS (58%) while only 1 of these AS cases (14%) is mosaic with respect to sSMC presence [44]. As the corresponding syndromes were caused either by uniparental disomy or microdeletion the sSMC presence has no direct influence on the clinical outcome; neither has mosaicism.

## SOMATIC MOSAICISM IN sSMC PRESENT IN STRUCTURALLY ABNORMAL BUT BALANCED KARYOTYPE

Another rare cytogenetic variant of sSMC presence is that of a structurally abnormal but balanced karyotype (McClintock mechanism) [48]. Such cases can either be connected with a neocentromere formation (see section below) or both the derivatives share the available centromeric alpha-satellite sequences. If in such case mosaicism appears, i.e. loss of the sSMC, relevant genetic material is lost and this leads normally to clinical problems as described for the following cases: 03-W-p11/1-1, 04-W-p15.3/1-1, 04-W-p12/1-1 [44]. If no or only very low grade mosaicism is present the carrier of such a karyotype can be completely normal (e.g. 02-O-p12/1-1, 06-O-p22.3/1-1, 06-O-p22.3/1-1, 08-O-p11.1/2-1, 12-U-4, 17-O-p11.2/2-1, mother of 19-W-10/2-1, mother of 22-W-q11.2/2-1 [44]). 

## SOMATIC MOSAICISM IN NEOCENTRIC sSMC

For mosaicism in neocentric sSMC formed by McClintock mechanism, [48] the same holds true, like for the aforementioned centric sSMC present in structurally abnormal but balanced karyotype. If balanced and no or only minimal mosaicism is present, the carriers of such a chromosomal condition are clinically normal. If the neocentric sSMC is lost in a higher percentage of the body cells this has an adverse prognosis.

In general, in at least around 50% of the cases with a neocentric sSMC somatic mosaicism is observable (Table **4**). Strikingly, as in centric sSMC, mosaicism is more frequent in non-acrocentric derived compared to acrocentric derived ones (58% vs. 24%). 

## SOMATIC MOSAICISM IN sSMC PRESENT IN NUMERICALLY ABNORMAL BASIC KARYOTYPES

As above mentioned, sSMC can appear in a numerically normal basic karyotype of 46 chromosomes, but also in numerically abnormal basic karyotypes [42]. Up to now, sSMC are reported additionally to a basic karyotype 45, X (= Turner syndrome), 47, XXY (= Klinefelter syndrome), 47, XXX (triple-X syndrome) and 47, +21 (Down syndrome) [44, 49-51].

542 cases are available in the literature with a basic karyotype typical for Turner syndrome and an additional sSMC, i.e. 46,X,+mar [44, 49]. Only 73 of these are reported without mosaicism; thus, 76% of these Turner syndrome cases are mosaic [44].

Only three cases, each of them are known by now with Klinefelter- or triple-X syndrome and an additional sSMC. Concerning the Klinefelter-syndrome two cases of those are mosaic (07-U-6, 0X-U3) and one not (09-U5) [44]. For triple-X syndrome the same holds true: cases 09-U16 and 14-O-q11.1/1-5 are mosaic, case 14-U-5 is not [44]. 

For sSMC, at present additionally to a trisomy 21 (Down-syndrome), information on mosaic status is available in 16 cases; 7 of those (44%) have somatic mosaicism with a cell line 47, +21 without sSMC [44]. 

Overall, mosaicism is a frequent finding when an sSMC is present additionally to a numerically abnormal basic karyotype. 

## SOMATIC MOSAICISM IN sSMC AND THE RESULTING PITFALLS

Summarizing all above mentioned groups, 1626 of 3124 cases with sSMC (52%) present with somatic mosaicism. Even though, expressed to a different extent in various sub-groups, mosaicism is something to be considered in at least every second such case. However, if a specific genetic imbalance caused by an sSMC is known to be harmful, in the overwhelming majority of the cases there is no influence of the grade of somatic mosaicism detectable in peripheral blood or amnion cells and the observed clinical effects. This seems to be due to the fact that the mosaicism rate in different human tissues is practically not predictable and very variable [52]. Only in exceptional cases the presence of specific sSMC with known adverse prognosis was reported which did not lead to clinical problems due to low somatic mosaicism; examples are 07-W-p10/1-1, 15-O-q13/1-1, 15-O-q13/1-2, 15-O-q13/2-1, 15-O-q13/3-1, 15-O-q13.1/1-1, 22-O-q11.21/4-2, 22-O-q11.21/4-3, 22-O-q11.21/5-1 [44]. Even though rare, this knowledge is extremely important for prenatal counseling.

Knowing that somatic mosaicism happens in ~50% of the cases with sSMC, array-CGH studies cannot be offered as a screening test to reliably detect this kind of chromosomal aberration. On the one hand, low level mosaic cases and on the other hand, cryptic mosaics are missed. Thus, cytogenetics is still the gold-standard to detect any kind of chromosomal aberration, which then, in further steps can be characterized by molecular (cyto-) genetic approaches. 

Interestingly, acrocentric and non-acrocentric derived sSMC are differently susceptible to mosaicism; acrocentric derived ones are hereby the more stable ones. This holds true for centric and neocentric sSMC, and an explanation is therefore at present not available. 

## CONCLUSION

Somatic mosaicism is a feature of the human body, which has to be considered much more than up to now in future. It is known as a fact, but not understood why man with age (in peripheral blood) develops something like a ‘Turner-syndrome-mosaic’ 46,XY/45,X. Similarly, in cases with sSMC it is known since years, that PKS patients lose the i(12p) in peripheral blood or that some inv dup(15) sSMC are stable and cytogenetically identical ones in another carrier are not. For all these facts to the best of our knowledge, no studies were undertaken to come closer to an understanding of these phenomena. Here we present, some kind of starting point for such studies, for the first time a detailed ‘mosaicism map’ for the different subtypes of sSMC.

## Figures and Tables

**Table 1:** Cases with Mosaics 47,+mar, Excluding Cases with Known Syndromes, with Neocentric sSMC and such with Unclear Mosaicism Status

sSMC derived from chromosome	Number of cases with 47,+mar[100%]	Total number of sSMC cases	Cases with mos 47,+mar/46
1	6	67	91%
1/5/19	0	8	100%
2	6	36	83%
3	7	21	67%
4	7	21	67%
5	10	34	71%
6	2	14	86%
7	5	23	78%
8	11	92	88%
9	4	59	93%
10	5	18	72%
11	3	16	81%
12	6	29	79%
13	7	9	22%
13/21	54	84	36%
14	62	99	37%
14/22	31	49	37%
15	361	459	21%
16	4	46	91%
17	6	26	77%
18	14	43	67%
19	7	40	83%
20	7	33	79%
21	12	25	83%
22	78	115	32%
acro	3	6	50%
X	7	27	74%
Y	6	13	54%
**overall**	**731**	**1512**	**52%**
**acrocentric**	**608**	**846**	**28%**
**non-acrocentric**	**123**	**666**	**82%**

**Table 2:** Cases with Cryptic Mosaics 47,+mar, Excluding Cases with Known Syndromes, with Neocentric sSMC and such with Unclear Mosaicism Status

sSMC derived from chromosome	Number of cases with cryptic mosaicism	Number of cases with cryptic mosaicism
1	0/67	0%
1/5/19	0/8	0%
2	2/36	5%
3	5/21	24%
4	1/21	5%
5	5/34	15%
6	4/14	29%
7	4/23	17%
8	8/92	9%
9	6/59	10%
10	0/18	0%
11	4/16	25%
12	2/29	7%
13	2/9	15%
13/21	0/84	0%
14	2/99	2%
14/22	0/49	2%
15	4/459	1%
16	3/46	19%
17	0/26	0%
18	2/43	5%
19	6/40	15%
20	4/33	12%
21	2/25	8%
22	5/115	4%
acro	0/6	0%
X	2/27	7%
Y	0/13	0%
**overall**	**73/1512**	**5%**
**acrocentric**	**15/846**	**2%**
**non-acrocentric**	**58/666**	**9%**

**Table 3:** Cases with Mosaics 48,+marx2 Excluding Cases with Known Syndromes, with Multiple and Neocentric sSMC and such with Unclear Mosaicism Status

sSMC derived from chromosome	Number of cases with 48,+marx2[100%]	Total number of sSMC cases	Cases with mosaic
1	n.a.	2 (diff. sizes)	100%
1/5/19	n.a.	n.a.	n.a.
2	n.a.	2	100%
3	n.a.	2 (diff. sizes)	100%
4	n.a.	1 / 1 (diff. sizes)	100%
5	n.a.	1 / 1 (diff. sizes)	100%
6	n.a.	1 / 1 (diff. sizes)	100%
7	n.a.	n.a.	n.a.
8	n.a.	2 / 1 (diff. sizes)	100%
9	n.a.	2 / 1 (diff. sizes)	100%
10	n.a.	n.a.	100%
11	n.a.	n.a.	n.a.
12	n.a.	n.a.	n.a.
13	1	1	0%
13/21	1	1 / 1 (diff. sizes)	0%
14	2	3 / 1 (diff. sizes)	50%
14/22	1	3	67%
15	11	22	50%
16	n.a.	1 / 1 (diff. sizes)	100%
17	n.a.	1 / 1 (diff. sizes)	100%
18	n.a.	n.a.	n.a.
19	1	1	0%
20	n.a.	3 / 1 (diff. sizes)	0%
21	n.a.	1	100%
22	n.a.	2	100%
acro	n.a.	n.a.	n.a.
X	n.a.	1	100%
Y	n.a.	1	100%
**overall**	**17**	**64**	**73%**
**acrocentric**	**16**	**35**	**54%**
**non-acrocentric**	**1**	**29**	**97%**

**Table 4:** Mosaicism in Cases with Neocentric sSMC

sSMC derived from chromosome	Number of cases with mosaicism	Percentage of cases with mosaicism
1	3/5	60%
2	3/4	75%
3	10/11	91%
4	1/1	100%
5	0/1	0%
6	1/2	50%
7	1/1	100%
8	7/9	77%
9	1/3	33%
10	1/2	50%
11	0/2	0%
12	2/3	75%
13	5/14	56%
14	1/1	100%
15	2/19	11%
16	1/1	100%
17	0/1	0%
18	1/1	100%
19	n.a.	n.a.
20	0/1	0%
21	n.a.	n.a.
22	n.a.	n.a.
X	0/1	0%
Y	0/1	0%
**overall**	**40/86**	**47%**
**acrocentric**	**8/34**	**24%**
**non-acrocentric**	**32/55**	**58%**
